# A pragmatic randomized controlled trial to evaluate the efficacy and safety of an oral short-course regimen including bedaquiline for the treatment of patients with multidrug-resistant tuberculosis in China: study protocol for PROSPECT

**DOI:** 10.1186/s13063-024-07946-9

**Published:** 2024-04-01

**Authors:** Jingtao Gao, Mengqiu Gao, Jian Du, Yu Pang, Gary Mao, Nacer Lounis, Nyasha Bakare, Yanxin Jiang, Ying Zhan, Yuhong Liu, Liang Li, on behalf of the Trial Team, Liu Rongmei, Du Juan, Wu Guihui, Pei Yi, Sha Wei, Shi Lian, Wang Hua, Jin Long, Wu Yuqing, Xiong Yu, Yan Xiaofeng, Chen Xiaohong, Huang Zhongfeng, Ren Fei, Li Xiujie, An Huiru, Cui Junwei

**Affiliations:** 1 Clinical Center On TB, Beijing Chest Hospital, Capital Medical University/Beijing Tuberculosis & Thoracic Tumor Research Institute, Beijing, People’s Republic of China; 2 Department of Tuberculosis, Beijing Chest Hospital, Capital Medical University/Beijing Tuberculosis and Thoracic Tumor Research Institute, Beijing, 101149 People’s Republic of China; 3 Department of Bacteriology and Immunology, Beijing Chest Hospital, Capital Medical University/Beijing Tuberculosis & Thoracic Tumor Research Institute, Beijing, 101149 People’s Republic of China; 4 Janssen Global Public Health, Janssen Research & Development, Titusville, NJ USA; 5 Janssen Pharmaceutica, Beerse, Belgium; 6 Janssen China Research & Development, Shanghai, People’s Republic of China; 7 Innovation Alliance On Tuberculosis Diagnosis and Treatment (Beijing) [IATB], Beijing, 101100 People’s Republic of China

**Keywords:** Tuberculosis, Bedaquiline, China, Oral Short-course regimen

## Abstract

**Introduction:**

The lack of safe, effective, and simple short-course regimens (SCRs) for multidrug-resistant/rifampicin-resistant tuberculosis (MDR/RR-TB) treatment has significantly impeded TB control efforts in China.

**Methods:**

This phase 4, randomized, open-label, controlled, non-inferiority trial aims to assess the efficacy and safety of a 9-month all-oral SCR containing bedaquiline (BDQ) *versus* an all-oral SCR without BDQ for adult MDR-TB patients (18–65 years) in China. The trial design mainly mirrors that of the “Evaluation of a Standardized Treatment Regimen of Anti-Tuberculosis Drugs for Patients with MDR-TB” (STREAM) stage 2 study, while also incorporating programmatic data from South Africa and the 2019 consensus recommendations of Chinese MDR/RR-TB treatment experts. Experimental arm participants will receive a modified STREAM regimen C that replaces three group C drugs, ethambutol (EMB), pyrazinamide (PZA), and prothionamide (PTO), with two group B drugs, linezolid (LZD) and cycloserine (CS), while omitting high-dose isoniazid (INH) for confirmed INH-resistant cases. BDQ duration will be extended from 6 to 9 months for participants with *Mycobacterium tuberculosis*-positive sputum cultures at week 16. The control arm will receive a modified STREAM regimen B without high-dose INH and injectable kanamycin (KM) that incorporates experimental arm LZD and CS dosages, treatment durations, and administration methods. LZD (600 mg) will be given daily for ≥ 24 weeks as guided by observed benefits and harm. The *primary outcome* measures the proportion of participants with favorable treatment outcomes at treatment completion (week 40), while the same measurement taken at 48 weeks post-treatment completion is the *secondary outcome*. Assuming an *α* = 0.025 significance level (one-sided test), 80% power, 15% non-inferiority margin, and 10% lost to follow-up rate, each arm requires 106 participants (212 total) to demonstrate non-inferiority.

**Discussion:**

PROSPECT aims to assess the safety and efficacy of a BDQ-containing SCR MDR-TB treatment at seventeen sites across China, while also providing high-quality data to guide SCRs administration under the direction of the China National Tuberculosis Program for MDR-TB. Additionally, PROSPECT will explore the potential benefits of extending the administration of the 9-month BDQ-containing SCR for participants without sputum conversion by week 16.

**Trial registration:**

ClinicalTrials.gov NCT05306223. Prospectively registered on 16 March 2022 at https://clinicaltrials.gov/ct2/show/NCT05306223?term=NCT05306223&draw=1&rank=1 {2}.

**Supplementary Information:**

The online version contains supplementary material available at 10.1186/s13063-024-07946-9.

## Introduction

### Background and rationale {6a}

Multidrug-resistant and rifampicin-resistant tuberculosis (MDR/RR-TB) poses a significant challenge to global public health. Regrettably, between 2018 and 2021 a mere 648,953 patients across the globe started second-line treatment for this serious condition, a number amounting to just 43% of the 1.5 million 5-year target for 2018–2022 that was established during the United Nations high-level meeting on TB in 2018 [1]. To address this issue, there is an urgent need for research and innovation, one of the three pillars of the End TB Strategy [2], to facilitate the development of shorter, safer, and efficacious MDR/RR-TB treatments. Prior to 2016, second-line anti-TB injectable drugs and oral drugs are administered to MDR/RR-TB patients for 6 to 8 months as an integral part of a 20-month treatment regimen. However, despite ongoing efforts, treatment success rates for MDR/RR-TB patients have remained disappointingly low. Moreover, patients must grapple with a substantial pill burden, significant adverse events, and other issues that have led to unsatisfactory treatment compliance [3]. Tragically, mortality rates associated with this relentless disease have been reported to approach 15% [4]. Since its introduction in 2010, the use of the shorter-course “Bangladesh regimen” [5] has been observed to achieve a high relapse-free cure rate, prompting the WHO to recommend further exploration of such regimens under operational conditions [6–10]. In 2016, the WHO issued conditional recommendations to guide the use of a standardized shorter-course MDR/RR-TB treatment regimen or a modified version of it based on local treatment experiences, as informed by results of observational studies conducted in various Asian and African countries [3].

In 2019, the WHO published consolidated drug-resistant TB (DR-TB) treatment guidelines that included key changes made to MDR/RR-TB treatment protocols [11]. The revised guidance introduces a priority ranking-based classification scheme for available anti-TB treatment drugs. In this scheme, BDQ is recommended and prioritized for use as a core drug (Group A) in the longer-course MDR/RR-TB treatment regimen, with LZD also recommended for inclusion as a core drug. A third Group A drug, such as levofloxacin (LFX) or moxifloxacin (MFX), and at least one Group B drug, such as clofazimine (CFZ), cycloserine (CS), or terizidone (TZD), are recommended for use in the longer-course MDR/RR-TB treatment regimen to ensure that at least four potentially effective TB agents are administered at treatment initiation. As an additional modification, kanamycin (KM) and capreomycin (CM) were deprioritized as components of MDR/RR-TB treatment regimens [11]. Meanwhile, the “Chinese expert consensus of MDR-TB and RR-TB treatment” guidelines issued in October 2019 recommend the reprioritization of certain drugs, as based on WHO 2019 guidance recommending the incorporation of BDQ in both long-course regimens (LCRs) and SCRs used to treat MDR-TB patients [12].

In December 2019, the WHO issued a Rapid Communication [13] advocating the adoption of a shorter-course, all-oral, BDQ-containing regimen for MDR/RR-TB patients to replace the standard shorter-course injectables-based regimen. At the same time, the results of a study conducted in South Africa [14, 15] prompted the WHO to recommend transitioning away from the standard regimen. These revisions are incorporated within the current WHO consolidated guidelines issued in 2020 [16] along with guidelines for administering a shorter-course (9–12-month) all-oral BDQ-containing regimen for treatment of eligible MDR/RR-TB cases lacking both resistance to fluoroquinolones (FQs) and prior treatment histories of greater than 1-month’s duration to second-line TB drugs included in the shorter-course, all-oral regimen.

As access to newer and repurposed TB drugs increases, Chinese researchers are increasingly focused on developing shortened regimens with lower toxicity and non-inferior or better efficacy that eliminate the need for injectables. The “Pragmatic Randomized Controlled Trial to Evaluate the Efficacy and Safety of an Oral Short-course Regimen Including BDQ for the Treatment of Patients with Multidrug-resistant Tuberculosis in China” (PROSPECT) was designed to compare the safety and efficacy of a modified 40-week all-oral, BDQ-containing SCR MDR-TB treatment to corresponding performance indicators of a 40-week all-oral SCR without BDQ. The design of the new MDR-TB treatment regimen, which primarily incorporates key drugs of regimens B and C evaluated in the STREAM stage 2 study [6], was further modified based on recommendations of experts, as indicated in the 2019 “Chinese expert consensus of MDR-TB and RR-TB treatment” guidelines^12]^. For the experimental arm treatment, modifications were made to the original STREAM regimen C. In accordance with principles governing LCR design, the three group C drugs EMB, PZA, and PTO were replaced with the two group B drugs LZD and CS and high-dose INH, while BDQ treatment duration was increased from 6 to 9 months for participants who were still sputum culture-positive for *MTB* at week 16. For the control arm, an 18–20-month injectables-based regimen, the recommended standard of care (SOC) MDR-TB treatment since 2016 [17], was replaced with the WHO-endorsed shorter-course 9–11-month injectables-based regimen in 2018 [18]. However, modified standardized shorter-course MDR-TB regimens are currently being developed for Chinese MDR-TB patients. For example, the PROSPECT control arm receives a modified treatment, whereby KM is replaced with LZD to remove the need for injectables that are no longer recommended by the WHO. Additionally, high-dose INH was removed, due to concerns stemming from (1) the high *KatG* mutation rate observed in INH-resistant *MTB* isolates obtained from patients in China, which implies that most Chinese TB cases with high-level INH resistance will not benefit from high-dose INH; (2) the small proportion of Chinese MDR-TB patients who carry a certain *ihnA* mutation who may benefit from high-dose INH treatment should be excluded, due to PTO cross-resistance conferred by this mutation; and (3) the safety profile of high-dose INH when used to treat Chinese patients has not been verified. In summary, the PROSPECT experimental arm modifications were made based on the original STREAM regimen C and the control arm regimen was modified based on the regimen B used in the STREAM study [6], by removing high-dose INH and injectable KM and intensified by incorporating LZD and CS.

The original PROSPECT protocol was submitted to regulatory officials on Mar 26, 2020. Two versions of the protocol were subsequently amended on Mar 9, 2021, and Dec 8, 2021, as requested by the China Center for Drug Evaluation (CDE). As a post-marketing approval commitment study, the results of PROSPECT are expected to provide updated evidence-based guidance for improving MDR-TB treatment practices in China.

## Objectives {7}

### Primary objectives

To evaluate the performance of an oral BDQ-containing SCR at treatment completion by comparing its efficacy to that of an oral SCR without BDQ in participants with pulmonary MDR-TB in China.

### Secondary objectives

- To compare favorable treatment outcomes of an all-oral BDQ-containing SCR versus those of an all-oral SCR without BDQ at 48 weeks post-treatment completion in participants with pulmonary MDR-TB in China.
- To evaluate efficacy based on treatment success of an all-oral BDQ-containing SCR versus an all-oral SCR without BDQ at treatment completion in participants with pulmonary MDR-TB in China.
- To evaluate the safety of an all-oral BDQ-containing SCR versus that of an all-oral SCR without BDQ in participants with pulmonary MDR-TB in China.
- To evaluate modified favorable treatment outcomes associated with the administration of an all-oral BDQ-containing SCR versus outcomes of an all-oral SCR without BDQ at treatment completion and at 48 weeks post-treatment completion in participants with pulmonary MDR-TB in China.
- To evaluate TB relapse and re-infection rates during the post-treatment follow-up phase in participants with pulmonary MDR-TB in China.
- To evaluate rates of emerging resistance to BDQ and other anti-TB drugs incorporated within the all-oral SCR with and without BDQ regimen used to treat participants with pulmonary MDR-TB in China.

## Trial design {8}

This is an open-label, parallel-group, randomized, controlled, multicenter, interventional, pragmatic, phase 4, clinical study that assesses non-inferiority. The study aims to assess the efficacy and safety of an oral BDQ-containing SCR as compared to corresponding performance indicators of an oral SCR without BDQ. Our goal is to enroll a total of 212 participants (106 participants/regimen) aged 18 to 65 years with microbiologically confirmed pulmonary MDR-TB. The study will be implemented within the framework of the China Tuberculosis Clinical Trial Consortium (CTCTC) network [19].

**Fig. 1:**
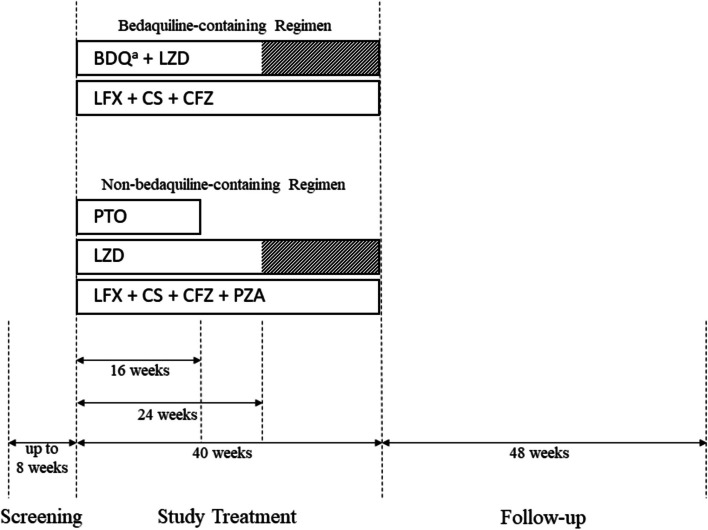
Schematic overview of the study design. BDQ, bedaquiline; CFZ, clofazimine; CS, cycloserine; LFX, levofloxacin; LZD, linezolid; PTO, prothionamide; PZA, pyrazinamide. ^a^If a participant is still sputum culture-positive for *Mycobacterium tuberculosis* by week 16, BDQ treatment will be extended from 24 to 40 weeks

The PROSPECT study process consists of a screening phase of up to 8 weeks, a study treatment phase of 40 weeks, and a 48-week follow-up phase occurring after treatment completion. Monitoring of all recruited participants will be conducted for 88 weeks post-randomization after completion of the screening phase. A diagram outlining the study design is provided in Fig. 1.

Assessments are scheduled at regular intervals according to the type of drug administered (Table 1), which aligns with local SOC practices. Efficacy assessments conducted at all sites will include sputum smears, sputum cultures performed using the Mycobacteria Growth Indicator Tube (MGIT) 960 system, drug susceptibility testing (DST) in cases with positive sputum cultures, and genotyping/fingerprinting of *MTB* isolates obtained from relapse or reinfection cases.

**Table 1:** Assessment schedule for patients recruited{13}

	**Study period**																
**Phase**	**Screening**	**Study Treatment**												**Follow-up**			
**Timepoint**	**W-8 to D-1**	**D1/BSL**	**W2**	**W4**	**W8**	**W12**	**W16**	**W20**	**W24**	**W28**	**W32**	**W36**	**W40**	**W52**	**W64**	**W76**	**W88**
**Enrolment:**																	
Screening																	
Informed consent	X																
Inclusion/exclusion criteria	X																
Chest CT	X					X			X			X					
Medical history and demographics	X																
Pregnancy test	X	X				X							X				
HIV test	X																
Symptoms/weight	X	X	X	X	X	X	X	X	X	X	X	X	X	X	X	X	X
Physical examination	X	X	X	X	X	X	X	X	X	X	X	X	X	X	X	X	X
Randomization		X															
**Interventions:**																	
Dispense/administer allocated TB treatment		X	X	X	X	X	X	X	X	X	X	X	X				
**Assessments:**																	
Study treatment adherence assessment			X	X	X	X	X	X	X	X	X	X	X	X			
Efficacy and safety evaluations																	
Sputum smear	X	X	X	X	X	X	X	X	X	X	X	X	X	X	X	X	X
Sputum culture	X	X	X	X	X	X	X	X	X	X	X	X	X	X	X	X	X
Molecular DST	X																
Collect strains for phenotypic DST (only in case of positive sputum culture)	X	X	X	X	X	X	X	X	X	X	X	X	X	X	X	X	X
Visual acuity testing	X	X	X	X	X	X	X	X	X	X	X	X	X				
Urinalysis	X	X	X	X	X	X	X	X	X	X	X	X	X				
Hematology	X	X	X	X	X	X	X	X	X	X	X	X	X				
Clinical chemistry^a^	X	X	X	X	X	X	X	X	X	X	X	X	X				
Hepatitis B and C testing		X															
TSH^b^	X	X				X			X								
ECG^c^	X	X	X	X	X	X	X	X	X	X	X	X	X				
Ongoing participant review																	
Concomitant therapy	X	X	X	X	X	X	X	X	X	X	X	X	X	X	X	X	X
AEs	X	X	X	X	X	X	X	X	X	X	X	X	X	X	X	X	X

X indicates assessments required at particular visits. *BSL* Baseline, *CT* Computerized tomography, *D* Day, *W* Week

^a^Will include liver and kidney function tests and electrolytes and blood glucose measurement

^b^Will be conducted at screening for all subjects; subsequent TSH testing will be conducted only if participant is receiving PTO

^c^ECG measurement should be continued during follow-up for patients whose bedaquiline course is extended to 40 weeks duration, and as clinically indicated to follow patients with increases in QTcF at the end of treatment until resolution

Safety evaluations will include regular physical examinations, monitoring of adverse events (AEs), visual acuity testing, urinalysis testing, electrocardiograms (ECGs), and routine blood testing to assess levels of hematological, chemical, and hepatitis B and C markers and levels of thyroid-stimulating hormone (TSH) for PTO-treated participants. In addition, hepatitis B and C status will be checked at baseline.

## Methods: participants, interventions, and outcomes

### Study setting {9}

The trial is sponsored by the Innovation Alliance on Tuberculosis Diagnosis and Treatment, Beijing (IATB) and conducted at Beijing Chest Hospital, Capital Medical University, and 16 other TB-specialized hospitals nationwide that are equipped to provide trial participants with both ambulatory and hospital-based care. Site details are available on the ClinicalTrials website (https://register.clinicaltrials.gov/prs/app/template/Home.vm?uid=U000369X&ts=39&sid=S000BTRA&cx=-t8d5e0.). The study protocol was drafted in accordance with the Standard Protocol Items: Recommendations for Interventional Trial guidelines (SPIRIT) [20].

### Eligibility criteria {10}

#### Inclusion criteria

A prospective participant must satisfy all of the following criteria to be enrolled in the study:

**Table:** 

1	Is willing and able to provide written informed consent (signed or witnessed consent if the participant is illiterate) to participate in study-related treatment and follow-up care activities
2	Tests positive for *MTB* via sputum culture during the screening period
3	Has microbiologically confirmed RIF resistance as determined via GeneXpert and *katG* mutation-induced resistance to INH, as revealed via molecular DST
4	Has chest imaging findings supporting a diagnosis of pulmonary TB
5	Is a man or woman aged 18 to 65 years
6	Agrees to use effective contraception during the 40-week study treatment phase; a non-vasectomized male participant must agree to use condoms during the 40-week study treatment phase
7	Has normal serum K^+^, Mg^2+^, and corrected Ca^2+^ levels at screening
8	Is willing to undergo HIV testing
9	Resides in close proximity to the study site and is able to remain under observation for the duration of the study

#### Exclusion criteria

Any potential participant who meets any of the following criteria will be excluded from participating in the study:

**Table:** 

1	Is infected with a strain of *MTB* with molecular DST-confirmed resistance to FQs, previously tested positive via phenotypic DST for resistance to any drug in the regimen or tested positive for low-level INH resistance that was either confirmed by phenotypic DST or through molecular detection of the *inhA* mutation
2	Has received prior BDQ treatment
3	Had prior exposure to at least one second-line drug in the regimen for at least 4 weeks
4	Is known to be pregnant, is planning to become pregnant, or is breastfeeding
5	Is unable or unwilling to comply with study treatment protocols or follow-up schedules
6	Has a grade three or grade four laboratory abnormality based on Division of AIDS (DAIDS) grading, as confirmed by a clinical expert
7	Has a blood ALT/AST ratio that is greater than three times the upper limit of normal (ULN) or a total blood bilirubin level greater than two times the ULN (participants with Gilbert’s disease will not be excluded as long as the total blood bilirubin level is < 2 × ULN); creatinine clearance of < 30 mL/min; blood hemoglobin level of ≤ 7.0 g/dL; blood platelet count of < 50 × 10^9^/L at screening
8	Is taking any medication contraindicated with drugs in the regimen
9	Has a known allergy or intolerance to BDQ or other drugs in the regimen
10	Is currently taking part in another study of a medicinal product
11	Has a QTcF of ≥ 450 ms at screening
12	Has a history of Torsade de Pointes or a history of cardiac risk factors for Torsade de Pointes, including a personal or family history of long QT syndrome, ongoing hypothyroidism, heart failure, or bradycardia (as defined by a sinus rate of < 50 bpm)
13	Has a history of syncopal episodes (i.e., cardiac syncope not including syncope due to vasovagal or epileptic causes) or symptomatic or asymptomatic arrhythmias
14	Has concomitant severe disease affecting cardiovascular, hepatic, renal, neurological, hematopoietic, or other systems or concomitant neoplastic disease(s)
15	Has uncontrolled diabetes mellitus
16	Has a concomitant mental disorder and, in the judgment of the investigator, is not fit for the study or unlikely to complete the full course of the study
17	Is HIV-positive
18	Is critically ill and, in the judgment of the investigator, is not fit for inclusion in the study or is unlikely to complete the full course of the study
19	Is infected with a strain of nontuberculous mycobacteria
20	Has isolated extrapulmonary disease
21	Has extrapulmonary disease that is miliary in nature or extrapulmonary disease involving the central nervous system and/or the skeletal system

### Who will take informed consent? {26a}

Each participant must provide written consent according to local requirements after the nature of the study has been fully explained to them. Written informed consent is provided by signing the informed consent form (ICF) before participation in any study-related activity. The ICF must be approved by both the sponsor/designee and by the reviewing IEC/IRB and written in a language that the participant can read and understand. Informed consent should adhere to principles as outlined in the Declaration of Helsinki, current ICH and GCP guidelines, applicable regulatory requirements, and sponsor/designee policies.

Before a potential participant is enrolled in the study, the investigator or an authorized study-site staff member must explain to him/her the aims, methods, reasonably anticipated benefits and potential hazards of the study, and any discomfort associated with study participation. Participants will be informed that their participation is voluntary, that they may withdraw consent to participate in the study at any time, and that choosing not to participate will not affect their care. Finally, participants will be informed that the investigator will maintain a participant identification register for use in providing long-term follow-up care (as needed) and that their records may be accessed by health authorities and authorized sponsor/designee personnel without violating participant confidentiality to the extent permitted by applicable laws or regulations. By signing the ICF, the participant is authorizing such access.

The participant will be given sufficient time to read the ICF and then will be provided an opportunity to ask questions. Thereafter, consent should be appropriately recorded as the participant’s personally dated signature on the ICF prior to study participation. After participant consent is obtained, a copy of the signed ICF must be given to the participant. A participant who is rescreened for study suitability is not required to sign another ICF if rescreening occurs within 30 days from the previous ICF signature date.

If the participant is unable to read or write, an impartial witness should be present during the entire informed consent process (which includes reading and explaining all written information) and should personally date and sign the ICF after the participant oral consent is obtained.

### Additional consent provisions for collection and use of participant data and biological specimens {26b}

Separate consent forms are needed for ancillary studies.

### Interventions

PROSPECT is a registration-related study to provide evidence of BDQ-containing SCR in Chinese based on STREAM. The experimental arm is modified based on STREAM regimen C (an oral regimen consisting of BDQ, LFX, CFZ, EMB, and PZA administered for 40 weeks, supplemented by high-dose INH and PTO in the first 16 weeks) and the control arm is modified according to STREAM regimen B (an injectable-containing regimen consisting of MFX or LFX, CFZ, EMB, and PZA administered for 40 weeks, supplemented by KM or AM, high-dose INH, and PTO in the first 16 weeks).

#### Explanation for the choice of comparators {6b}

The SCR without BDQ consists of LFX (40 weeks), LZD (at least 24 weeks), CS (40 weeks), CFZ (40 weeks), pyrazinamide (PZA) (40 weeks), and PTO (initial 16 weeks) and is designed to serve as a comparator under self-administration trained by appointed nurses The control arm treatment is modified by removing high-dose INH and injectable KM, then regimen efficacy is enhanced by adding LZD and CS based on Regimen B of STREAM trial.

#### Intervention descriptions {11a}

Experimental arm participants will be instructed to self-administer the assigned dose of BDQ in combination with the following four drugs: LFX (40 weeks), LZD (at least 24 weeks), CS (40 weeks), and CFZ (40 weeks). During the study treatment phase, these participants will receive an all-oral SCR consisting of 24 weeks of BDQ in combination with 40 weeks of other allocated TB treatment drugs (except for LZD, which will be administered for at least 24 weeks). If a participant is still sputum culture-positive for *MTB* by week 16, BDQ treatment will be extended from 24 to 40 weeks.

Detailed information for drugs administered to trial participants is provided in Table 2.

**Table 2:** Detailed information for the individual drug of each arm in the PROSPECT study

Drug	BDQ	LFX	LZD	CS	CFZ	PZA	PTO
BDQ-containing regimen (duration [weeks])	X (24 to 40)^a^	X (40)	X (≥ 24)	X (40)	X (40)		
Non-BDQ-containing regimen (duration [weeks])	NA	X (40)	X (≥ 24)	X (40)	X (40)	X (40)	X (First 16)
Dosage formulation	Uncoated tablet	Film-coated tablet	Tablet	Capsule	Capsule	Tablet	Enteric-coated tablet
Unit dose strength	100 mg	100 mg and 500 mg	600 mg	250 mg	50 mg	250 mg	100 mg
Dose regimen	400 mg once daily for the first 2 weeks, 200 mg 3 times per week (with at least 48 h between doses) for the remaining weeks (i.e., 22 weeks or 38 weeks in case bedaquiline treatment is extended to 40 weeks)	30–35 kg: 600 mg daily 36–55 kg: 750 mg daily > 55 kg: 1000 mg daily	600 mg daily	30–45 kg: 500 mg daily > 45 kg: 750 mg daily	100 mg daily	30–35 kg: 1000 mg daily 36–70 kg: 1500 mg daily > 70 kg: 2000 mg daily	30–45 kg: 500 mg daily 46–70 kg: 600 mg daily > 70 kg: 800 mg daily

^a^If a participant is still sputum culture-positive for *MTB* by week 16, BDQ treatment will be extended from 24 to 40 weeks

*NA*, not available

#### Criteria for discontinuing or modifying allocated interventions {11b}

A participant’s study intervention must be discontinued if:

- The participant withdraws consent to receive the study intervention.
- The investigator believes that for safety or tolerability reasons it is in the best interest of the participant to discontinue the study intervention. Individual drug substitutions within the study intervention regimen may be considered if clinically indicated at the discretion of the investigator. Administration of single-drug substitutions will not be viewed as an unfavorable treatment outcome criterion if limited to substitutions of non-group A drugs (except DLM) as per the 2020 WHO MDR-TB treatment guidelines.
- The participant becomes pregnant.
- Unacceptable toxicity.
- The patient experiences:
  - Aminotransferase elevations with total bilirubin elevations to levels of ≥ twofold the ULN
  - Aminotransferase elevations to levels that are ≥ eightfold greater than the ULN
  - Aminotransferase elevations to levels that are ≥ fivefold greater than the ULN and persist for more than 2 weeks.
- If the patient experiences a QTcF interval > 500 ms (as confirmed by repeat ECG) and/or clinically significant ventricular arrhythmia.
- Noncompliance with study drug administration.
- Any participant not meeting eligibility criteria as specified in the “Eligibility criteria” section.

A participant will be withdrawn from the study for any of the following reasons:

- If phenotypic DST results from a screening sample become available post-baseline confirming resistance at the start of the study to any of the drugs in the assigned regimen except BDQ, in which case the patient will be treated according to local treatment protocols after withdrawal from the study.
- Lost to follow-up.
- Withdrawal of consent.
- Death.

#### Strategies to improve adherence to interventions {11c}

BDQ and other allocated TB treatment drugs will be administered orally after screening either in a hospital or home setting according to site protocols. When participants are dosed in the hospital, the date of each dose will be recorded in the source documents and case report forms (CRFs) by designated study-site personnel. The study intervention dose, allocated TB treatment drugs, and study participant identifier will be confirmed at the time of dosing by a study-site staff member other than the person administering the study intervention.

For participants who self-administer the study intervention at home, patient compliance with the study intervention protocol will be assessed at each visit by counting remaining tablets/capsules during site visits and documenting this information in source documents and CRFs. Deviations from the prescribed dosage regimen should be recorded in the CRFs. A record of the number of tablets/capsules dispensed to and consumed by each participant must be maintained and reconciled with study intervention and compliance records. Intervention start and stop dates, including dates for intervention delays and/or dose reductions, will also be recorded in CRFs.

#### Relevant concomitant care and interventions that are permitted or prohibited during the trial {11d}

##### Concomitant therapy

Pre-study therapies administered up to 30 days prior to administration of the first dose of a study intervention must be recorded at the time of screening. Concomitant therapies must be recorded throughout the study from the time of initiation of the first study intervention dose.

All therapies, including prescription or over-the-counter medications (e.g., vaccines, vitamins, and herbal supplements) and non-pharmacologic therapies (e.g., electrical stimulation, acupuncture, special diets, exercise regimens, or other specific categories of interest) that differ from study interventions, must be recorded in the CRFs. Recorded information will include a description of the type of therapy, duration of use, dosing regimen, route of administration, and indication. Modification of an effective preexisting therapy should not be made for the explicit purpose of entering a participant into the study.

##### Prohibited medications

The use of the following drugs is not permitted concomitantly with BDQ administration through day 30 after administration of the last dose of BDQ:

- Systemic use of moderate and strong cytochrome P450 3A4 (CYP3A4) inhibitors for more than three consecutive days.
- Systemic use of moderate or strong CYP3A4 inducers.
- Medications of the statin class of compounds.
- Tricyclic antidepressants, including amitriptyline, doxepin, desipramine, imipramine, and clomipramine.
- Nonsedating antihistamines astemizole and terfenadine.
- Phenothiazine neuroleptic drugs thioridazine, haloperidol, chlorpromazine, trifluoperazine, percycline, prochlorperazine, fluphenazine, sertindole, and pimozide.
- The prokinetic drug cisapride.
- Quinoline antimalarials and quinolones used for other indications.
- Gatifloxacin and MFX.

#### Provisions for post-trial care {30}

If a participant discontinues a study intervention for any reason before the end of the study treatment period, end-of-intervention and post-intervention assessments will be conducted and scheduled study intervention assessments will continue during the 48-week follow-up phase, as outlined in the protocol. Participants who discontinue treatment are encouraged to complete quarterly visits for 1 year as per the protocol schedule unless they withdraw informed consent. Documentation of assessment results obtained after treatment discontinuation is encouraged. Based on patient treatment responses, investigators will formulate and administer more suitable new treatment regimens as needed. Investigational drugs will not be incorporated into new treatment regimens administered to patients.

### Outcomes {12}

#### Primary outcomes

The primary outcome is the proportion of participants with a favorable treatment outcome at treatment completion (week 40). In this work, patient outcomes will be classified as favorable if their last three culture results prior to treatment completion are negative, unless they had previously been classified as unfavorable. These three cultures must be taken on separate visits, the latest of which will be taken no more than 8 weeks prior to the treatment completion date. Unfavorable outcomes include the following: treatment discontinuation; starting 2 or more drugs not in the allocated regimen; treatment continuation beyond the permitted duration; death; lost to follow-up prior to treatment completion; relapse and re-infection. Treatment success is achieved if participants completed their prescribed TB treatment or if their last three culture results by the end of treatment are negative (these 3 cultures must be taken on separate visits).

#### Secondary outcomes

- Proportion of participants with a favorable treatment outcome at 48 weeks post-treatment completion.
- Proportion of participants achieving treatment success at treatment completion (week 40).
- Proportion of participants experiencing mortality from all causes.
- Proportion of participants experiencing grade 3 or greater treatment-emergent adverse events (TEAEs) during study treatment and follow-up phases.
- Proportion of participants experiencing TEAEs of all types.
- Proportion of participants with a modified favorable treatment outcome at treatment completion and 48 weeks post-treatment completion.
- Proportion of participants with TB relapses and re-infections during the post-treatment follow-up phase.
- Proportion of participants harboring *MTB* that develops resistance to BDQ and other drugs included in the regimen.

### Participant timeline {13}

See Table 1.

### Sample size {14}

The primary study null hypothesis posits that the all-oral, BDQ-containing SCR is inferior to that of the all-oral SCR without BDQ. Conversely, the primary study alternative hypothesis posits that the all-oral, BDQ-containing SCR is non-inferior to that of the all-oral SCR without BDQ. Both hypotheses will be tested by measuring the intergroup difference in the proportion of patients achieving favorable treatment outcomes at treatment completion, with results of analysis interpreted using a 15% non-inferiority margin and a 2.5% one-sided significance level.

Under the null hypothesis of inferiority, the favorable treatment outcome rate at treatment completion is assumed to be 85% for the control SCR and 70% for the BDQ-containing regimen. To achieve 80% power in demonstrating the non-inferiority of the BDQ-containing SCR as compared to the control SCR using the abovementioned non-inferiority margin and significance level cutoffs, a sample size of 95 evaluable patients per regimen is required.

Assuming that 10% of randomized patients would not be evaluable for the primary treatment outcome analysis, a total of 212 randomized patients (106/regimen) would be needed.

### Recruitment {15}

Patients who have received recent GeneXpert results indicating resistance are offered counseling sessions and may be considered for inclusion in the screening phase after providing informed consent. To inform the public about the study, news of its initiation is published in local newspapers, and both electronic and printed posters are distributed at each site to recruit participants.

Healthcare staff have established patient-centered care groups to offer consultation, health education, and psychological support throughout the study. On average, we are admitting eight to 10 cases per month at the current rate. Additionally, we have prepared new trial sites in case recruitment progresses slower than planned.

## Assignment of interventions: allocation

### Sequence generation {16a}

Participants who meet the enrollment criteria will be randomly assigned in a 1:1 ratio to the BDQ-containing SCR or the SCR without BDQ. The randomization schedule will be balanced using permuted blocks and stratified by study site and extent of lung cavitation (no cavity or cavity < 2 cm, presence of at least one cavity ≥ 2 cm in one lung only, presence of at least one cavity ≥ 2 cm in both lungs). The randomization sequence is generated by SAS 9.4.

### Concealment mechanism {16b}

The subject randomization schedule will be generated by the randomization statistician and imported into the Interactive Web Response System (IWRS) by the system engineer.

### Implementation {16c}

Randomization is available only to eligible participants who have provided the required randomization information, including stratified factors. The IWRS will then assign a randomization number to participants in sequential order based on the pre-established randomization table. Each randomization number will correspond to a specific group (intervention group or control group). The allocation sequence is prepared by an independent randomization statistician and the investigator of each site will enroll participants, and the interventions will be assigned by IWRS according to the production list.

## Assignment of interventions: blinding

### Who will be blinded {17a}

PROSPECT is an open-label study.

### Procedure for unblinding if needed {17b}

No unblinding procedure is needed for PROSPECT because it is an open-label study.

## Data collection and management

### Plans for assessment and collection of outcomes {18a}

Efficacy assessments included sputum smears, sputum cultures performed using the MGIT 960 system at all sites, DST of isolates derived from *MTB*-positive sputum cultures, and *MTB* genotyping/fingerprinting. Safety assessments will include regular physical examinations, monitoring of AEs, visual acuity testing, and routine blood examinations (e.g., hematology, clinical chemistry, TSH level testing for subjects receiving PTO, urinalysis, and ECGs). All study data will be recorded in source documents and transcribed by study-site personnel from source documents to generate electronic case reports (eCRFs) using an electronic data capture (EDC) system (https://prospect.imeddig.com.). To ensure that recorded data are consistent with original source data, source documents will be mutually agreed upon by the sponsor and investigators at each site before they are made available for direct access.

### Plans to promote participant retention and complete follow-up {18b}

Patients will be educated about the importance of treatment adherence with an emphasis on self-administration of scheduled drugs and completion of clinical follow-up visits. To reduce the chance that participants will be lost to follow-up, attempts will be made to obtain contact information (e.g., home, work, and mobile telephone numbers and email addresses) from each participant and appropriate family members. A participant will not be deemed lost to follow-up until all reasonable efforts have been made by study-site personnel to contact the participant. The following actions must be taken if a participant fails to return to the study site for a required study visit:

- Study-site personnel must attempt to promptly contact participants to reschedule missed visits. During this communication, they should counsel the participant on the importance of adhering to the assigned visit schedule and ascertain whether the participant wishes to or should continue to participate in the study.
- Before a participant is deemed lost to follow-up, the investigator or designee must make every reasonable effort to reestablish contact with the participant. Such efforts should include conducting a minimum of three telephone calls, sending emails and/or fax communications, and if warranted, sending a certified letter to the participant’s last known mailing address (or local equivalent method). Every attempt to make contact should be recorded in the participant’s medical records. In the event that the participant cannot be contacted, the participant will be officially categorized as having voluntarily withdrawn from the study.

### Data management {19}

All eCRFs will be designed and generated by the sponsor/designee in accordance with protocols and requirements as stipulated by the Code of Federal Regulations Title 21, Part 11 (21 CFR Part 11). To maintain data integrity, all eCRF entries, corrections, and alterations will be exclusively carried out by the investigator or authorized study-site personnel who have received relevant training in accordance with eCRF Completion and Data Entry Guidelines. The investigator assumes the vital responsibility of verifying the accuracy and correctness of all eCRF data entries. Following participant visits, eCFRs will be completed as soon as possible to ensure that completed forms are readily accessible for review during the subsequent scheduled monitoring visit. For consistency and standardized reporting, investigators will record adverse events as coded terms derived verbatim from the Medical Dictionary for Regulatory Activities (MedDRA).

The data management team (DMT) will conduct regular reviews to ensure data completeness and accuracy in accordance with the Data Management Plan and Data Management Quality Control Plan. The EDC system will perform logical checks automatically after data entry using a built-in algorithm that aligns with the data validation plan (DVP). Manual data checks will be performed monthly and may increase in frequency with rising data input and database locking rates. Both logical and manual review queries will be generated using the EDC system to request clarifications for inaccurate or incomplete data. Database locking will adhere to preestablished rules as indicated in the corresponding standard operating procedure (SOP).

### Confidentiality {27}

The collection, sharing, and processing of personal data from participants enrolled in this study are limited to those data that are necessary to fulfill study objectives. Such data must be collected and processed with adequate precautions to ensure confidentiality and compliance with applicable data privacy protection laws and regulations. Appropriate technical and organizational measures will be taken to ensure that participant personal data will not be subject to unauthorized disclosures or access, accidental or unlawful destruction, or accidental loss or alteration. Sponsor/designee personnel whose responsibilities require access to personal data will be required to agree to maintain patient confidentiality. A unique trial number will be assigned to each participant.

### Plans for collection, laboratory evaluation, and storage of biological specimens for genetic or molecular analysis in this trial/future use {33}

Three sputum samples will be collected from each participant at pre-scheduled visits, as detailed in Table 1; these samples will include one sample taken at night, one collected early the next morning, and one coached spot expectoration sample. While awaiting receipt of phenotypic DST results, participant *MTB* isolates will be screened for RMP resistance using GeneXpert, while other molecular DST methods will be used to screen for FQs resistance and INH resistance-associated *inhA* and *katG* mutations. Participants harboring *MTB* with susceptibility to RMP and INH, FQ resistance, or *inhA* mutation-induced INH resistance will be excluded from the trial. Participants harboring *MTB* with RMP resistance, INH sensitivity, or high-level INH resistance due to *KatG* mutations and FQs susceptibility, as confirmed using molecular methods, may be enrolled in the study while awaiting phenotypic DST results.

MGIT-based DST will be performed on cultures derived from specimens obtained at baseline, during treatment, and during the follow-up period. Phenotypic DST will be performed during the screening phase to detect resistance to RMP, INH, LFX, LZD, CFZ, PTO, and BDQ. If sputum cultures obtained at screening test positive for drug resistance within 1 month prior to the date of administration of the first dose of the study regimen, the culture obtained at screening may serve as the baseline culture. Post-baseline cultures of specimens collected from participants during or after week 16 of treatment that test positive for *MTB* will be stored until assayed via DST. DST will also be performed on any *MTB*-positive culture derived from specimens obtained during the follow-up period. Cultures obtained from specimens collected during the follow-up period from participants with treatment success who later revert to *MTB*-positive status (as indicated by culture conversion or disease relapse) will be genotyped, with paired *MTB*-positive isolates used for genotypic analysis.

## Statistical methods

### Statistical methods for primary and secondary outcomes {20a}

Primary efficacy analysis will be conducted using a stratified analysis method, whereby the risk difference for each stratum is determined using Cochran Mantel–Haenszel weights. Using this analysis method, differences in favorable treatment outcome rates at the end of treatment between the control SCR and the BDQ-containing SCR will be estimated along with corresponding 95% confidence intervals (CIs) and *p*-values. Thereafter, intergroup rate differences will be stratified according to study site and lung cavitation status. To meet pre-specified non-inferiority criteria, the upper bound of the 95% CI of the difference in the proportion of the favorable treatment outcome must be < 15%. Primary efficacy analysis will be conducted using the efficacy analysis dataset, as defined in Table 3. For the secondary outcomes, point estimates and CI values will be calculated to estimate differences in proportions between the BDQ-containing SCR and the control SCR without BDQ using the same stratified analysis as for the primary endpoint.

**Table 3:** Populations set for analysis

Population	Description
Intent-to-treat (ITT)	All randomized participants
Safety analysis set	All randomized participants who consume at least 1 dose of the study intervention
Modified ITT	Subset of the ITT with proper diagnosis, including all randomized participants with a positive *MTB* culture result at screening or randomization; excludes participants who provided isolates prior to randomization that were subsequently found to harbor RIF-susceptible *MTB* or participants who provided isolates prior to randomization that were subsequently found to be resistant to drugs in the regimens (including low-level INH resistance), as revealed by phenotypic DST results
Efficacy analysis set	All enrolled participants with confirmed pulmonary MDR-TB and a positive *MTB* culture at screening or baseline who were not withdrawn from the study because they harbored *MTB* with baseline resistance to prescribed drugs and consumed at least 1 dose of study treatment

### Interim analyses {21b}

Interim analysis is not planned for data obtained in this study.

### Methods for any additional analyses (e.g., subgroup analyses) {20b}

Subgroup analysis will be performed according to demographic variables, such as study site, lung cavitation, gender, age, drug resistance profile, and comorbidities.

### Methods in analysis to handle protocol non-adherence and any statistical methods to handle missing data {20c}

We plan to perform an intention-to-treat analysis and will address missed data by analyzing available data or by employing multiple imputation techniques.

### Plans to give access to the full protocol, participant-level data, and statistical code {31c}

The full protocol will be accessible after publication. Participant-level data will be accessible after the main results are published, with access to the completely encrypted dataset granted, as appropriate, by the corresponding author.

## Oversight and monitoring

### Composition of the coordinating center and trial steering committee {5d}

The steering committee (SC) is responsible for overseeing all trial-related activities and resource allocation at both the project level and site level to ensure that trial objectives and targets are met according to the established schedule. The SC approves the protocol and any modifications, while also supervising the activities of the DMT and the project management team (PMT). The DMT performs tasks related to eCRF design, EDC system validation, data review, and data reconciliation, while the PMT translates the project strategic measures and objectives set by the SC into clinical trial operational practices related to the implementation of routine technical, financial, and administrative duties.

### Composition of the data monitoring committee, its role, and reporting structure {21a}

A data monitoring committee will not be utilized for this study. Instead, periodic monitoring visits will be conducted by ClinChoice (https://clinchoice.com/.), a contracted research organization (CRO). The CRO’s designee will review study data for accuracy, completeness, and consistency by comparing the data to that indicated on the source document during on-site monitoring visits and after transmission to the sponsor. Any discrepancies will be resolved by the investigator or designee as needed.

### Adverse event reporting and harms {22}

AEs in this study are defined and reported using the following five general categorical descriptors, as defined in the Division of AIDS (DAIDS) Table for Grading the Severity of Adult and Pediatric Adverse Events, version 2.1, which was published in 2017 [21]. Participants (or, as appropriate, their caregivers, surrogates, or legally acceptable representatives) will be responsible for reporting AEs for the entire duration of the study. All AEs, whether serious or non-serious, will be reported from the moment a signed and dated ICF is obtained until completion of the participant’s final study-related procedure and follow-up safety assessment.

Every AE, regardless of seriousness, severity, or presumed relationship with the study intervention, must be documented using medical terminology in source documents and CRFs. When signs and symptoms are attributed to a common cause, diagnoses should be provided whenever possible. Investigators must record their opinions concerning the relationships of AEs to study therapies in the CRFs. All measures taken to manage AEs must be recorded in the source document and reported according to sponsor/designee instructions.

An AE is categorized as serious if it leads to death, permanent or significant disability, a congenital anomaly, or birth defect; is life-threatening; or necessitates hospital admission for management. Follow-up monitoring of all AEs detected during the trial will be conducted and documented until AE resolution or improvement occurs. All serious AEs (SAEs) occurring during the study must be promptly reported to the sponsor/designee contact person by study-site personnel within 24 h of detection of the event using designated SAE forms. SAEs that are unexpected or deemed related to any drug used in the trial will be reported to the National Medical Products Administration (NMPA) and local Institutional Review Boards (IRBs).

### Frequency and plans for auditing trial conduct {23}

The sponsor/designee will perform on-site study monitoring visits as needed, as directed by the monitoring plan that was prepared prior to study initiation. Monitors will regularly meet with investigators to provide feedback on study performance. Initial monitoring will focus on site evaluation; the second round of monitoring will be conducted as soon as possible after initiation of enrollment and will be followed by regular site monitoring visits occurring at a frequency dictated by recruitment speed. During these visits, direct access to source documents (medical records) must be allowed for the purpose of verifying that recorded data are consistent with original source data. Findings from this monitoring will be discussed with the study-site personnel.

The sponsor/designee expects study-site personnel to be available during monitoring visits, that source documents will be accessible, and that a suitable environment will be provided for document review. The final monitoring visit will be conducted during site closeout. Additionally, external audits may be conducted by the sponsor and/or NMPA inspections may occur.

### Plans for communicating important protocol amendments to relevant parties (e.g., trial participants, ethical committees) {25}

All protocol amendments must be issued by the sponsor/designee and signed and dated by the investigator. Protocol amendments must not be implemented without prior approval of the Research Ethics Committee (REC) and/or IRB or when the relevant competent authority has raised concerns that may lead to non-acceptance, except when such amendments are necessary to eliminate immediate hazards to participants. In such exceptional cases, the amendment must be promptly submitted to the REC/IRB and relevant competent authority. Documentation of amendment approval by the investigator and REC/IRB must be provided to the sponsor/designee. For changes limited to logistical or administrative aspects of the study, notification to the REC/IRB (where required) suffices. Both the original protocol and any subsequent amendments must be submitted to the appropriate regulatory authorities, and the study cannot commence until all local regulatory prerequisites are met.

### Dissemination plans {31a}

Results of the study will be submitted for publication in relevant scientific and peer-reviewed journals. The preliminary results will be presented at global academic conferences.

## Discussion

The development of new anti-TB drugs and the use of repurposed drugs have paved the way for global explorations of novel drug combinations and treatment durations [16, 22, 23]. In recent years, numerous innovative shorter regimens incorporating various drug combinations have been evaluated for efficacy under programmatic, operational research, or trial conditions [24–39]. BDQ was approved by NMPA in 2016 but it was commercially available until 2020. Before 2020, BDQ was only available under the umbrella of a new anti-TB drug introduction and prevention project (NDIP) for operational research and only 1513 MDR-TB patients were covered. Due to the relatively high price of BDQ, it was gradually widely used until recent years benefiting from the implementation of local-specific medical reimbursement policy.

In 2022, WHO updated consolidated guidelines on DR-TB treatment [40] wherein it recommended the use of a 6-month regimen based on BDQ, PA824, and LZD (BPaL) in combination with MFX (BPaLM), as based on results obtained in the TB-PRACTECAL trial [33, 37]. In addition, the WHO recommends the use of 6-month regimens incorporating BPaL in combination with a lower dose or shorter treatment duration of LZD, as based on favorable treatment outcomes obtained for both regimens in the ZeNix study [36]. However, PA824 is not approved by NMPA until now, so it is not available in China, thus limiting the exploration of the efficacy of this simpler, shorter, and patient-friendly regimen in the country. In PROSPECT, neither MFX nor DLM is administered in combination with BDQ to Chinese patients, due to the previously reported relatively high QTcF prolongation rate (24.7%) and 3.1% experienced post-baseline QTcF ≥ 500 ms in Chinese patients treated with BDQ in combination with MFX and other drugs [41]; notably, the latter rate exceeds the global average rate as described in the first global active drug safety monitoring report [42]. Nevertheless, administering DLM with BDQ has been shown to exert at most an additive QTcF prolongation effect [43], warranting further study. Meanwhile, results of the STREAM Stage 2 study [39] demonstrated that both a 9-month oral BDQ-containing regimen and a 6-month BDQ-containing regimen incorporating injectables provided superior favorable treatment outcome rates of 83% and 9%, respectively, as compared to that of a 9-month regimen containing injectables without BDQ (71%). Given the fact that the regimen administered to the control arm in our study contained more effective drugs (LZD and CS) than those included in Regimen B that was administered to the control arm in STREAM [6], it is estimated that higher rates of favorable outcomes will be obtained in both the control and experimental arms of PROSPECT. Furthermore, low rates of acquired drug resistance to each key drug administered to both arms during STREAM stage 2 were observed, with an extremely low BDQ resistance rate observed [39].

Interestingly, a similarly designed regimen to that administered to the PROSPECT experimental arm was evaluated in the NExT study [29], a randomized trial of a 6-month oral five-drug regimen developed in South Africa. This regimen included BDQ, LZD, and LFX, as well as two group B or C drugs that were selected based on the detection of mutations conferring *MTB* resistance as follows: high-dose INH will be selected for *inhA* mutation, ethionamide for *katG* mutation, or TZD for both *inhA* and *katG* mutations. Notably, NExT intervention arm participants were 2.2 times more likely to experience favorable outcomes at 24 months than participants receiving the 9-month WHO-approved injectable-based regimen [51% (25/49) versus 22.7% (10/44); RR 2.2 (1.2–4.1); *p* = 0.006]. Surprisingly, results of the recent Korean MDR-END study [26, 27] demonstrated that a new all-oral 9-month regimen consisting of DLM, LZD, LFX, and PZA was non-inferior to the conventional 20- to 24-month regimen in participants with FQ-sensitive MDR-TB, thus paving the way for investigations of additional SCR regimens incorporating DLM or combinations of BDQ with DLM. Additionally, a cost-effectiveness sub-study of PROSPECT is currently underway to generate cost data for BDQ-containing SCRs and SCRs without BDQ that will enable cost comparisons to be made between the two regimens.

Our study design had several limitations. First, the control arm did not receive the WHO-endorsed SOC regimen, which was designed based on STREAM regimen B, a regimen that adheres to the MDR-TB drug hierarchy as indicated in the WHO consolidated guidelines. Instead, the control arm received a modified regimen tailored to align with current treatment practices in China. Second, the open-label design of the PROSPECT study may introduce bias into our results that might be regarded as a limitation. Nonetheless, it was not possible to run the trial using a double-blinded design, due to different numbers of drugs in control and intervention arms and prolonged BDQ treatment of patient subpopulations with persistently *MTB*-positive sputum cultures at week 16. However, the statistician will be blinded throughout the duration of the study to prevent the introduction of additional bias into our results. Third, PROSPECT eligibility criteria barred patients younger than 18 years of age or older than 65 years of age from participating in the study, as well as those who are HIV-positive, afflicted with RR-TB, pregnant, and/or critically ill, thus potentially limiting the generalizability PROSPECT results. Fourth, MFX and DLM are not included in PROSPECT regimens, due to the relatively high occurrence of QT interval prolongation in Chinese patients after exposure to these drugs [41, 44] and thus were not evaluated in this study, despite their effectiveness as MDR-TB treatments in other populations. Last but not least, the primary design of the study, which was initiated in early 2019, was based on current knowledge at that time. Nonetheless, unforeseen delays prolonged the completion of CDE-required protocol review, modification, and approval processes until late 2021. Consequently, due to its status as a BDQ post-marketing commitment study in China, PROSPECT should conform to the approved protocol, although some study elements may be outdated when applied to global populations.

Safer, shorter, simpler, all-oral regimens for MDR/RR-TB are currently being explored globally, with the results of these efforts demonstrating that these drugs hold promise as effective MDR-TB treatments as indicated in the recently updated WHO guidelines. Nevertheless, translation of the knowledge obtained from these endeavors into clinical practices will be key to lowering treatment-related costs, reducing healthcare-related burdens, expanding treatment options, and improving treatment outcomes for patients suffering from MDR/RR-TB.

## Trial status

The trial is currently operating under protocol version 2.0, and the first participant was recruited on June 9, 2022, and recruitment will be completed at the early beginning of 2024.

### Supplementary Information

**Additional file 1.** SPIRIT Checklist for Trials.

## Data Availability

The data and materials will be available after the main results have been published.

## Abbreviations

SCRs: Short-course regimens
MDR/RR-TB: Multidrug-resistant/rifampicin-resistant tuberculosis
BDQ: Bedaquiline
STREAM: Evaluation of a Standardized Treatment Regimen of Anti-Tuberculosis Drugs for Patients with MDR-TB
EMB: Ethambutol
PZA: Pyrazinamide
PTO: Prothionamide
LZD: Linezolid
CS: Cycloserine
INH: Isoniazid
KM: Kanamycin
DR-TB: Drug-resistant
LFX: Levofloxacin
MFX: Moxifloxacin
CFZ: Clofazimine
TZD: Terizidone
CM: Capreomycin
LCRs: Long-course regimens
FQs: Fluoroquinolones
PROSPECT: Pragmatic Randomized Controlled Trial to Evaluate the Efficacy and Safety of an Oral Short-course Regimen Including BDQ for the Treatment of Patients with Multidrug-resistant Tuberculosis in China
SOC: Standard of care
CDE: Center for Drug Evaluation
CTCTC: China Tuberculosis Clinical Trial Consortium
MGIT: Mycobacteria Growth Indicator Tube
DST: Drug susceptibility testing
AEs: Adverse events
ECGs: Electrocardiograms
TSH: Thyroid-stimulating hormone
IATB: The Innovation Alliance on Tuberculosis Diagnosis and Treatment, Beijing
ULN: Upper limit of normal
ICF: Informed consent form
CRFs: Case report forms
CYP3A4: Cytochrome P450 3A4
TEAEs: Treatment-emergent adverse events
IWRS: The Interactive Web Response System
eCRFs: Electronic case reports
EDC: Electronic data capture
MedDRA: Medical Dictionary for Regulatory Activities
DMT: Data management team
DVP: Data validation plan
SOP: Standard operating procedure
CIs: Confidence intervals
PMT: Project management team
CRO: Contracted research organization
DAIDS: Division of AIDS
SAEs: Serious AEs
NMPA: The National Medical Products Administration
IRBs: Institutional Review Boards
ITT: Intent-to-treat
REC: Research Ethics Committee
BPaL: A 6-month regimen based on BDQ, PA824, and LZD
BPaLM: BPaL in combination with MFX
PA824: Pretomanid
